# Rapid prototyping-assisted tooth autotransplantation is associated with a reduced root canal treatment rate: a retrospective cohort study

**DOI:** 10.1186/s12903-022-02058-9

**Published:** 2022-02-02

**Authors:** Lisa Alice Hwang, Chi-Yuan Chang, Wei-Chia Su, Chi-Wha Chang, Chien-Yu Huang

**Affiliations:** 1 Department of Oral and Maxillofacial Surgery, Chia-Yi Chang Gung Memorial Hospital, Chia-Yi County, Taiwan; 2 Department of Stomatology, College of Medicine, National Cheng Kung University, Tainan, Taiwan; 3 Department of Oral and Maxillofacial Surgery, Kaohsiung Chang Gung Memorial Hospital, Kaohsiung City, Taiwan; 4 Department of Otolaryngology, Chia-Yi Hospital, Ministry of Health and Welfare, No. 312, Beigang Rd., West Dist, Chiayi City, 600 Taiwan; 5 Department of Otolaryngology Head and Neck Surgery, Kaohsiung Veterans General Hospital, Kaohsiung City, Taiwan

**Keywords:** Autotransplantation, Rapid prototyping, Root canal treatment

## Abstract

**Background:**

Autotransplantation is a beneficial treatment with a high success rate for young patients. However, most adult patients require root canal treatment (RCT) of the donor teeth after the autotransplantation procedure, which causes a prolonged treatment time and additional expenses and increases the rate of future tooth fracture. Rapid prototyping (RP)-assisted autotransplantation shortens the extra-alveolar time and enables a superior clinical outcome. However, no cohort studies of the application of this method on adult populations have been reported.

**Methods:**

This study is a retrospective cohort study. All patients underwent autotransplantation from 2012 to 2020 in the Kaohsiung and Chia-Yi branches of Chang Gung Memorial Hospital, and the procedure and clinical outcomes were analysed. Differences in clinical outcomes, age, sex, extra-alveolar time, fixation method, and RCT rate were compared between the two groups.

**Results:**

We enrolled 21 patients, 13 treated using the conventional method and 8 treated using the RP-based technique. The RCT rates of the conventional group and RP group were 92.3% and 59%, respectively. The mean age of the two groups was significantly different (28.8 ± 10 vs. 21.6 ± 2.1); after performing subgroup analysis by excluding all of the patients aged > 40 years, we found that the RCT rates were still significantly different (91.0% vs. 50%). The mean extra-alveolar time was 43 s in the RP group, and the autotransplantation survival rate in both groups was 100%.

**Conclusions:**

Rapid prototyping-assisted autotransplantation was successfully adopted for all patients in our study population. By shortening the extra-alveolar time, only 50% of the patients required a root canal treatment with a 100% autotransplantation survival rate.

*Trial Registration* : Retrospectively registered.

## Background

Autotransplantation is defined as the surgical extraction of a donor tooth and the transfer of that tooth to a receptor site in the same patient. It is a beneficial treatment option for tooth replacement, especially for younger patients, because it provides a vital periodontium and continual skeletal growth. In previous studies, success was defined as the direct physiological implantation of the donor tooth, without any signs of pathology or the need for additional procedures. Furthermore, survival was defined as the persistence of the transplanted tooth (despite possible compromised function, aesthetics, or development) [1]. A long-term follow-up study revealed a 90% survival rate in a cohort followed for 26.4 years, with a success rate of 79% [2]. Machado et al. [3] reported the long-term survival rate of autotransplantation to be 75%–91%. These studies mostly focused on teenage populations, and studies on adult autotransplantation are scarce. From a literature review, poor prognostic factors for tooth survival include patient age ˃45 years, mandibular location for the donor tooth, and extra-alveolar time ˃ 15 min, as reported by Jang et al. [4]. Aoyama et al. [5] reported the following poor prognostic factors for autotransplanted teeth: history of donor tooth root canal treatment (RCT), multirooted donor tooth, maxillary donor tooth, and duration of tooth absence at the recipient site. Of these, donor tooth extra-alveolar time is the sole variable that can be controlled by oral surgeons and improved with skill and proper equipment.

The key considerations for successful tooth transplantation are preservation of healthy periodontal ligament cells and favourable tissue adaptation. These goals are influenced by surgical factors such as the number of attempts to fit the donor tooth, distance between the new alveolus and the root of the donor tooth, extra-alveolar time, surgeon skill, and trauma level during donor tooth extraction [6, 7].

The advent of the combination of cone-beam computed tomography (CBCT) and rapid prototyping (RP) has permitted accurate simulation of the surgery target to aid in precise surgical planning. After its first reported clinical application in 1950 [8], the success rate of tooth autotransplantation has gradually increased due to advances in diagnostic and surgical techniques such as computer-aided RP (CARP) modeling. By applying preoperatively fabricated CARP models, extra-alveolar time is considerably reduced, and the suitability between the donor tooth and the recipient site is improved [9]. EzEldeen et al. [10] compared CARP-guided autotransplantation with the conventional method in a group of children with a mean age of 10–11 years and found that both the survival rate and success rate were superior in the CARP-guided group. Shahbazian et al. [11] published a case–control study of 40 paediatric patients with a mean age of 11 years, demonstrating that CARP-based surgical planning of autotransplantation may result in reduced surgical time, a less invasive technique, and fewer failures than conventional approaches. Verweij et al. [12] conducted a systematic literature review and concluded that no randomized controlled trial in the field of RP-assisted autotransplantation or case–control study on adult patients has been conducted for this method.

The aim of this study was to report our application of RP-assisted autotransplantation in adult patients (age ≥ 18 years old) who were at relatively high risk of failure because of age, already formed donor tooth roots and poorly viable periodontal ligament cells.

## Methods

### Study design and population

This study was reviewed and approved by the Chang Gung Medical Foundation Institutional Review Board (202100492B0). From February 2012 to October 2020, all patients who underwent tooth autotransplantation with a follow-up of > 6 months in our dental department were enrolled in this study. The inclusion criteria for autotransplantation were the following: rejection of implant placement with 1 nonretainable tooth, early tooth loss, a congenitally missing tooth in the premolar or molar region with a third molar or a malpositioned or impacted premolar and a suitable shape and dimension for the recipient site after clinical and radiographic evaluation. The exclusion criteria were poor oral hygiene, moderate to severe periodontitis, age > 55 years, or general contraindications for transplant surgery. The patient’s age, sex, donor site, recipient site, extra-alveolar time and procedure time were recorded. All patients signed an informed consent form for receiving the autotransplantation surgery. The surgery was conducted either by conventional method or RP methods based on the in charge surgeons’s preference for not all surgeons were familiar with the RP method. All autotransplantations using the RP-assisted technique were performed by one and the same experienced oral maxillofacial surgeon (Lisa H). Randomized control trial was not suitable in this study for considering the low incidence of tooth autotransplantation which may lengthen the study period and resulting time-dependent variability. No blinding was performed because of the evident difference in surgical approach.

### Tooth autotransplantation with conventional and RP methods

The conventional method of transplantation used an extracted donor tooth as a template for the preparation of the recipient site. The stiches were removed 2 weeks postoperation, and the wire splint was removed 1 month postoperation when mobility was downgraded to ≤ Gr. I. Vitality was checked with electric pulp testing every month; if vitality indicated a negative measurement and no apical bone healing was seen, RCT was arranged.

**Fig. 1:**
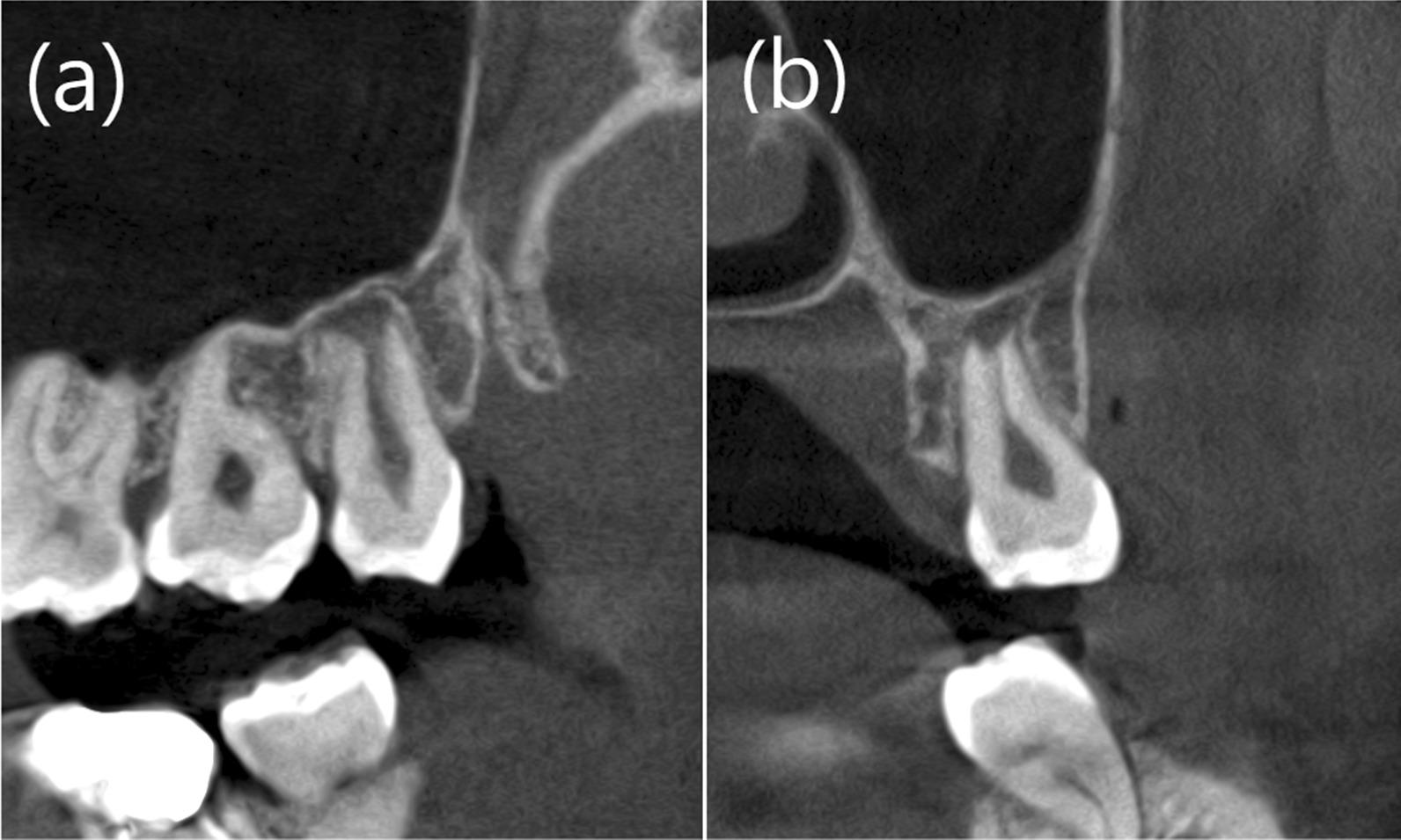
The Cone-Beam CT of example patient (Patient 2). **a** Sagittal view of the donor tooth 28. **b** Coronal view of the donor tooth 28

For the RP group, the Digital Imaging and Communications in Medicine data for the teeth were obtained from CBCT images (Fig. 1).

**Fig. 2:**
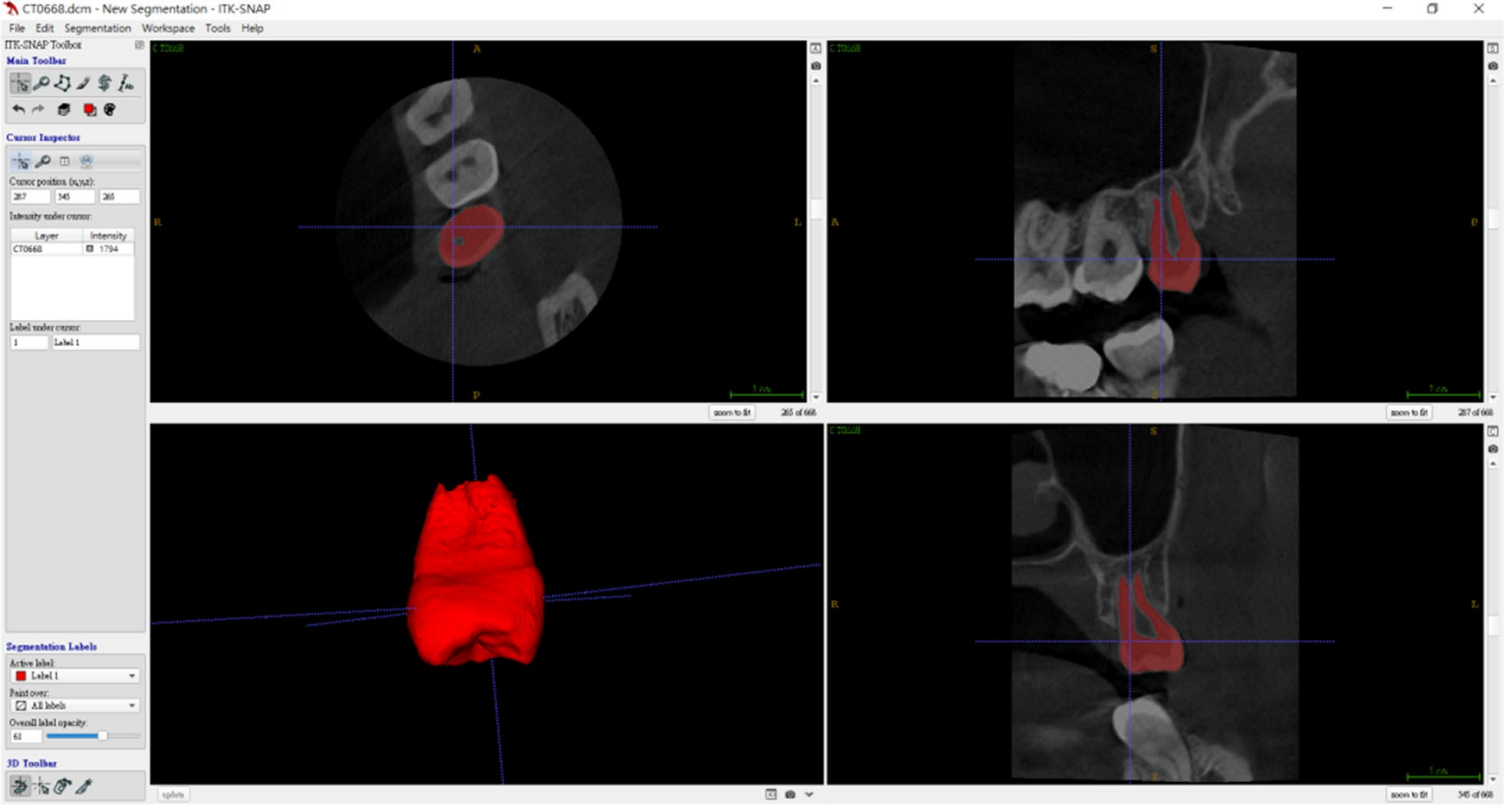
Constructing the surface mesh from the donor tooth Cone-Beam CT with the ITK-SNAP software

The images were loaded into ITK-SNAP software(version 3.6.0) [13] (a free open-source software developed by Penn Image Computing and Science Laboratory at the University of Pennsylvania and Guido Gerig, Ph.D., of the Scientific Computing and Imaging Institute at the University of Utah) for segmentation of the desired tooth and nearby jawbone. In the software, active contour segmentation method was chose with lower threshold set at 1400, then place 6–8 bubbles for optimizing segmentation and execute evolution till ideal mesh is constructed (Fig. 2).

**Fig. 3:**
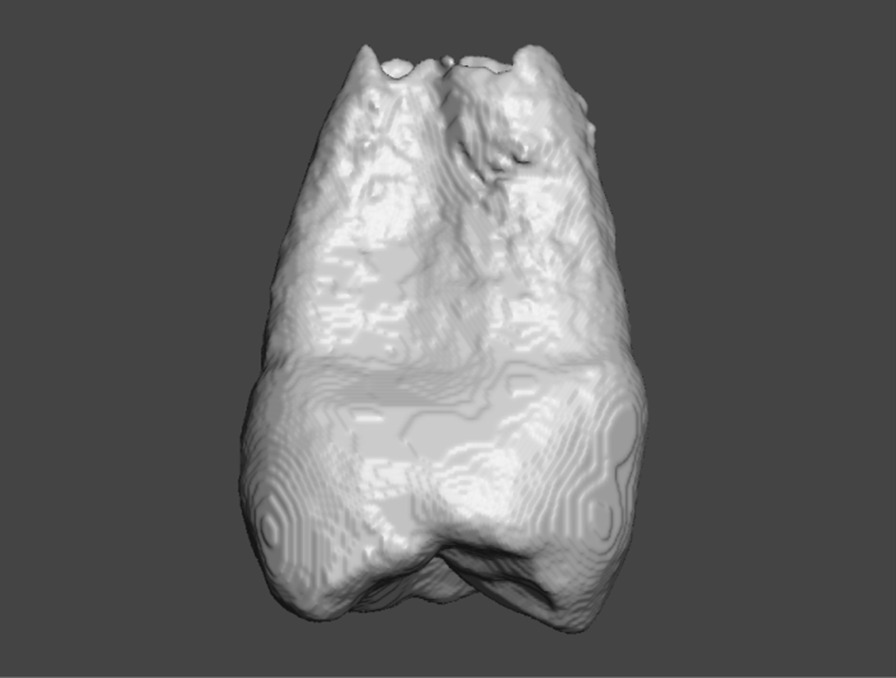
The STL file displayed in the Autodesk Meshmixer software for surface polishing

The mesh was subsequently saved as a binary stereolithography (STL) file, which was then imported into Autodesk Meshmixer (Autodesk, San Rafael, CA, USA) for mesh surface polishing (Fig. 3).

**Fig. 4:**
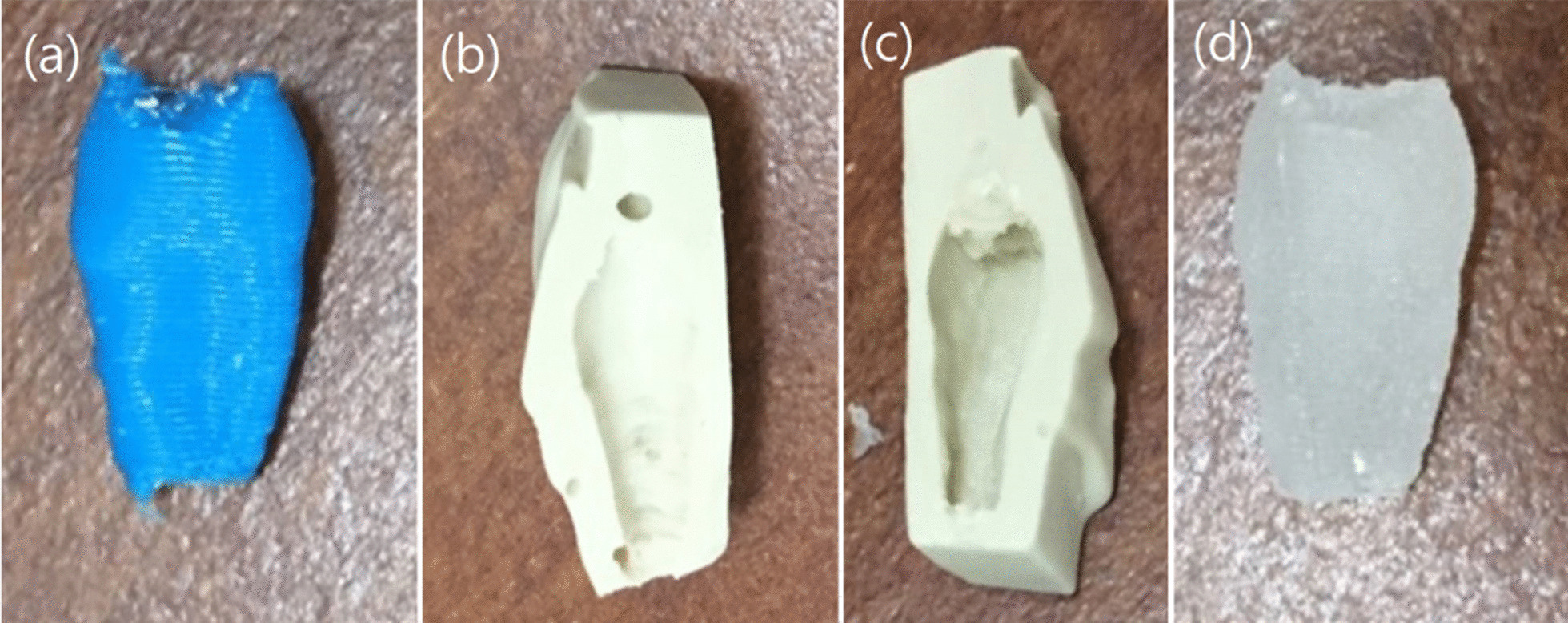
The workflow of biocompatible model fabrication. **a** The RP 3D-printed donor tooth model, **b**, **c** Negative mode made with alginate impression of the donor tooth(Cut in half). **d** Donor tooth replica made with Orthoresin from the negative mode

**Fig. 5:**
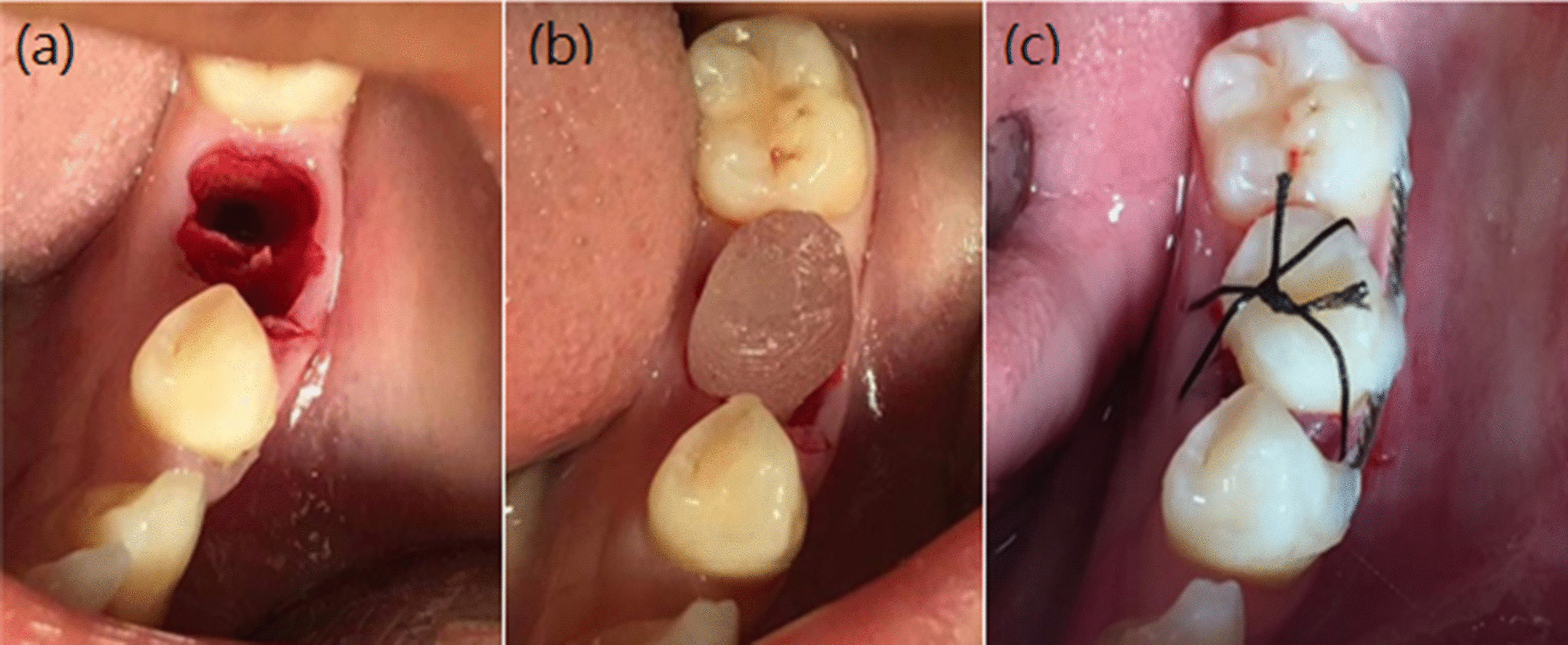
Procedures for fitting the Orthoresin replica into the recipient’s socket. **a** Donor site preparation with the aid of the 3D-printed Orthoresin replica. **b** The fitness of the donor tooth root was confirmed with the Orthoresin replica. **c** Fixation of donor teeth in the recipient’s socket

For further fabrication, we used fused filament fabrication, performing 3D printing on a FlashForge Creator Pro (FlashForge, Jinhua, Zhejiang, China), with high-resolution 3D-printed material, namely, 1.75-mm polylactic acid. Printing times varied depending on printing size and material density settings and ranged from 5 to 10 min. After obtaining the 3D model of the donor tooth, a negative model was made through alginate impression, and subsequently, the donor tooth replica was made with Orthoresin (Fig. 4), which was used as a surgical template after disinfection (Fig. 5).

### Statistical analysis

All statistical analyses were performed using SPSS (version 15; SPSS, Chicago, IL, USA). Continuous variables were analysed using one‐way ANOVA, and categorical variables were compared using Pearson’s chi‐square test or Fisher’s exact test. Two‐sided *P* < 0.05 was considered significant.

## Results

Eight patients underwent RP-assisted autotransplantation, and 13 underwent the conventional autotransplantation method. The characteristics of all of our RP-assisted autotransplantation patients are presented in Table 1.

**Table 1:** Characteristics of rapid prototyping assisted autotransplantation patients

Patient	Age (years)	Sex	Donor tooth	Recipient site	Fixation	Extra-Alveolar time (sec)	Follow up time (months)	Vitality
1	20	M	28	36	Wire + suture	65	13	Vital
2	21	F	28	36	Wire + suture	3	26	Vital
3	21	M	28	36	Wire	22	19	Vital
4	23	F	48	46	Wire + suture	120	40	RCT
5	18	M	18	16	Suture	58	12	Vital
6	22	M	18	37	Suture	30	12	RCT
7	24	M	18	47	Suture	25	17	RCT
8	24	F	24	45	Wire + suture	45	31	RCT

The mean age of our RP-assisted autotransplantation patients was 21.6 ± 2.1 years. Most donor teeth were the third molars. The recipient’s site was most commonly the first molar, followed by the second molar. The mean extra-alveolar time was 43 s, and all patients had a follow-up of ≥ 12 months. The survival rate was 100%, and 50% of patients required RCT (Table 1). The results of univariate analysis comparing RP-assisted autotransplantation and the conventional method are presented in Table 2.

**Table 2:** Comparison of rapid prototyping and conventional autotransplantation

	Rapid prototyping	Convention	*p* value
*All cases*			
Case number	8	13	
Age (yr)	21.6 ± 2.1	28.8 ± 10	0.025*
Sex (male %)	62.50%	23.10%	0.077
Root canal treatment(%)	50%	92.30%	0.027*
*Age < 40y/o cases*			
Case number	8	11	
Age (yr)	21.6 ± 2.1	25.7 ± 6.8	0.082
Sex (male %)	62.50%	27.30%	0.139
Root canal treatment (%)	50%	91.00%	0.048*

*Indicate *p* < 0.05 with statistical significance

**Fig. 6:**
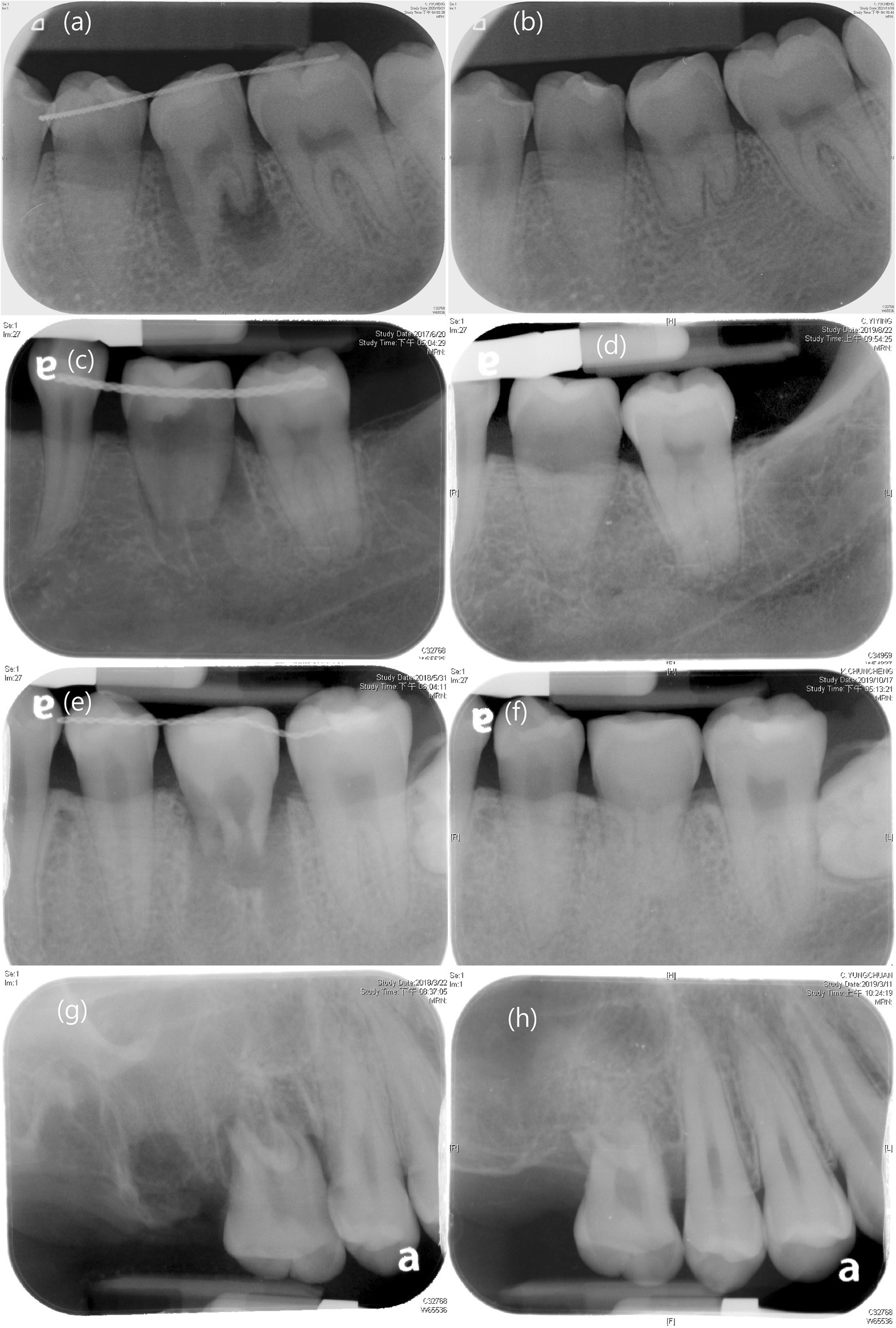
X-ray image of the transplanted tooth. **a** Patient 1, operation day. **b** Patient 1, 1 year after the operation. **c** Patient 2, operation day. **d** Patient 2, 2 years after the operation. **e** Patient 3, operation day. **f** Patient 3, 1.5 years after the operation. **g** Patient 5, operation day. **h** Patient 5, 1 year after the operation

Age and RCT rate were significantly different between the two groups; the conventional method group was older (28.8 ± 10 vs. 21.6 ± 2.1, *P* = 0.025). Thus, subgroup analysis was performed and excluded all patients aged > 40 years; subsequently, age was no longer significantly different (21.6 ± 2.1 vs. 25.7 ± 6.8, *P* = 0.082). However, in this subgroup, the RCT rate was still significantly lower in the RP-assisted group than in the conventional method group (50% vs. 91%, *P* = 0.048). Interestingly, in the RP group, we observed continuous root formation and clear PDL space in teeth that had not undergone RCT (Fig. 6).

## Discussion

Several published case reports and case series have demonstrated the benefit of RP-assisted tooth autotransplantation [6, 7, 9, 11, 12, 14, 15]. Most of them are case reports, but 1 is a case–control study analysing RP-assisted autotransplantation in paediatric patients. No randomized control trial comparing this novel method with the conventional method has yet to be conducted. To the best of our knowledge, this study is the first adult (age ≥ 18 years) retrospective cohort study to compare the outcome of using RP-assisted autotransplantation with that of the conventional method. By applying this technique, we found that adults, like their paediatric counterparts, could expect several benefits, namely, decreased extra-alveolar time and the RCT rate, and that continuous root formation is possible when the donor tooth remains vital even in adult patients.

To obtain a satisfying outcome, efforts must be made to perform minimally traumatic surgical extraction and reduce the extra-alveolar time to more adeptly preserve Hertwig’s epithelial root sheath and pulp vitality [16]. Xia et al. [14] reported that the average extra-alveolar time of their 28 CARP autotransplantation cases was 2.5 min. Kim et al. [6] reported a total of 182 autotransplantation cases using the CARP technique, with an average extra-alveolar time of 7.58 min. Shahbazian et al. [11] reported their experience in a case–control study that revealed an average extra-alveolar time of < 1 min for the study group but up to 3–10 min for the control group. Our study participants had an average extra-alveolar time of 46 s with a 100% survival rate, which provides more evidence of the usefulness of this technique.

Regarding the accuracy of the RP process, Shahbazian et al. [17] reported that the replica was accurate to within 0.25 mm of a native tooth. They concluded that this finding indicated the feasibility of applying stereolithographic models for in vivo planning of CBCT-based autotransplantation. Lee and Kim found that 3D CT images of teeth were on average 0.149 mm smaller in size than real teeth, whereas donor tooth replicas were on average 0.067 mm smaller in size than CT images of the teeth. Lee et al. [9] comparatively evaluated the accuracy of two distinct printing technologies—fused deposition modelling (FDM) and PolyJet. The FDM replicas were slightly smaller than the original donor teeth, but the PolyJet models were slightly larger. Although these distinctions were statistically significant, the authors regarded them as clinically nonsignificant. Khalil et al. [18] evaluated the accuracies of three 3D printing technologies—stereolithography, FDM, and PolyJet—and found that the differences between the dimensions of the teeth printed with these technologies and those of the original tooth were well below the clinically required surgical accuracy level of 0.25 mm.

The treatment options for missing teeth include single implantation, bridge fabrication, and tooth autotransplantation. Autotransplantation is not the first-line treatment option for adults with a single missing tooth for several reasons. First, due to the limited candidate teeth, autotransplantation usually requires younger patients with healthy third molars. Second, autotransplantation can yield relatively unpredictable outcomes versus implant and prosthetic bridge fabrication, and the transplanted tooth may need root canal treatment or experience ankylosis or root resorption. Third is financial considerations. In Taiwan, tooth autotransplantation is covered by Taiwan national health insurance, and the surgeon will receive 140 USD from the government for performing an autotransplantation procedure. If the patient agrees to the RP method, he or she must pay 350 USD out of pocket. However, in general, a 3-unit bridge requires a patient to pay 1700 USD, and a single implant costs 3000 USD. With the help of the RP method in autotransplantation, the success rate increases dramatically, and the operation time decreases relative to the conventional method. Autotransplantation has several benefits, including avoiding preparation of adjacent teeth, unlike bridge fabrication, and a shorter treatment time than implant treatment. This could be a valuable treatment option for young adults with a limited financial budget.

The present study had numerous strengths, including our precise measurements of the extra-alveolar time for each RP-assisted procedure, which yielded evidence of the usefulness of this technique. We provided a low-cost method for transforming an office-based 3D printing model into a disinfected, biocompatible tooth replica, which could make this technique more accessible. However, our study also had several limitations. First, this was a retrospective study, which may have led to selection bias, and our control group was significantly older than the experimental group. Thus, subgroup analysis was performed to exclude all patients aged > 40 years; consequently, the two groups had no remaining significant difference in age, but a difference in the RCT rate remained. Second, the follow-up time was relatively short, since we only started the RP-assisted autotransplantation method in 2017.

## Conclusions

The use of RP for autotransplantation enabled accurate positional planning and decreased the extra-alveolar time and RCT rate. With the assistance of RP, routine endodontic treatment of transplanted teeth may not be required.

## Data Availability

The datasets used and/or analysed during the current study are available from the corresponding author on reasonable request.

## Abbreviations

CBCT: Cone-beam computed tomography
RCT: Root canal treatment
CARP: Computer-aided rapid prototyping
RP: Rapid prototyping
STL: Stereolithography
